# Response to Incremental Replacement of Palm Oil with Fish Oil in Starter Diet on Growth Performance, Plasma Metabolites, Ruminal Fermentation, and Behavior of Dairy Calves

**DOI:** 10.3390/ani14071061

**Published:** 2024-03-30

**Authors:** Seyed Hadi Hosseini, Hamidreza Mirzaei-Alamouti, Morteza Mansouryar, Mina Vazirigohar, Pedram Rezamand, Ehsan Mahjoubi, Jafar Ramezankhani, Jörg R. Aschenbach

**Affiliations:** 1 Department of Animal Science, Faculty of Agriculture, University of Zanjan, Zanjan 45371-38111, Iran; hadihosseini@alumni.znu.ac.ir (S.H.H.);; 2 Institute of Veterinary Physiology, Freie Universität Berlin, Oertzenweg 19b, 14163 Berlin, Germany; mina.vazirigohar@fu-berlin.de; 3 Zist Dam Group, University Incubator Center, University of Zanjan, Zanjan 45371-38791, Iran; 4 Department of Animal and Veterinary Science, University of Idaho, Moscow, ID 83844-2330, USA

**Keywords:** fish oil, n-3 fatty acid, palm oil, weaning, calves

## Abstract

**Simple Summary:**

In this study, we aimed to understand how different levels of fatty acids in the starter feed affect the growth and health of milk-fed calves. We divided 30 female calves into three groups and fed them starter feeds supplemented with different fatty acids: palm fatty acids (PO), a mix of palm fatty acids and fish oil (PFO), and fish oil (FO). We found that calves receiving FO had slightly greater body weight over the whole period compared to those receiving PFO or PO. However, overall growth rates and intake of feed remained similar across all groups. Additionally, parameters like body size, rumen fermentation, blood composition, and behavioral patterns were not affected by the type of fatty acids in their diet. Our study suggests that altering the ratio of different unsaturated fatty acids in starter feed may not have a substantial impact on the growth and metabolic performance of young calves under normal conditions. These findings contribute to our understanding of calf nutrition and may guide future strategies for optimizing their health and growth during the critical pre-weaning period.

**Abstract:**

The objective of this study was to evaluate the effects of the incremental levels of n-3 fatty acids (FA) in starter feed (SF) on growth and metabolic performance of milk-fed calves. From day 3 of age, 30 female calves (39.4 ± 3.1 kg of body weight) were randomly assigned to one of three dietary treatments: (1) SF supplemented with 3.3% palm fatty acids (PO), (2) SF supplemented with 1.7% of PO and 1.9% fish oil (PFO), or (3) SF supplemented with 3.9% fish oil (FO). Chopped straw (7.5% of DM) was included in the SF of all treatments as total mixed ration (TMR). Diets had similar energy and protein contents. Total n-3 FA (% of total FA) and n-6/n-3 of PO, PFO, and FO were 1.90, 6.80, and 11.8 and 15.5, 4.50, and 2.70, respectively. The BW was greater for calves receiving FO (60.2 ± 0.3 kg) compared with PFO (58.7 ± 0.3 kg; *p =* 0.007) and tended to be greater for calves receiving FO vs. PO (59.0 ± 0.3 kg; *p =* 0.050). Because there was no interaction effect between diet × week of experiment, the greater BW of FO could not be attributed to the dietary treatment. Accordingly, average daily gain, total dry matter intake (DMI), starter DMI, and gain to intake ratio (G:FI) did not differ among dietary treatments during the entire period of the study (*p* > 0.05). Dietary treatments did not impact body size parameters such as body length, body girth, withers height, heart girth, hip height, and width (*p* > 0.05). Neither ruminal fermentation parameters nor blood variables were influenced by supplementing the types of oil at different time points. Calves’ behavioral parameters, such as standing, lying, eating, and ruminating, were not influenced by different dietary treatments (*p* > 0.05). The number of days with abnormal fecal score was not different among dietary groups (*p* > 0.05). Overall, our findings suggest that changing the n-6/n-3 ratio in starter feed by incremental replacement of palm fatty acid with fish oil at a moderate supplemental level of ~3% of DM may not affect the growth and metabolic performance of young calves under non-challenged conditions.

## 1. Introduction

The pre-weaning period is a critical stage for maximizing productivity and minimizing the risk of health issues in newborn calves. In this regard, appropriate nutritional approaches play a pivotal role. Additionally, the European Union’s ban on antibiotic growth promoters, which were widely used to enhance the performance and health of newborn animals, makes it even more crucial to find alternative approaches suitable for newborn livestock. In addition to being a rich source of energy, fatty acids (FAs), especially n-3 poly- unsaturated FAs, serve various biological functions, including provision of anti-inflammatory or pro-resolving precursors [1]. Calves are born with low reserves of FAs [2], and their tissues contain low amounts of n-3 fatty acids because ruminant diets (high-grain diets) generally contain low concentrations of n-3 FAs compared to n-6 FAs. Therefore, adding appropriate amounts of n-3 FAs by including, e.g., fish oil in the diet of calves may be a valuable nutritional strategy to resolve inflammation [1] and improve their performance.

Hill et al. [3,4,5] demonstrated that adding a blend of FAs, including polyunsaturated FAs (PUFAs), to the starter feed (SF) or milk replacers could enhance growth performance parameters while reducing scouring and respiratory disease occurrence rates in young calves. Panahiha et al. [6,7] found that supplementing the starter diet with palm fatty acids (saturated fatty acid source) compared to soybean oil (n-6 FA source) had superior effects on the performance of young calves before weaning. It is well documented that FAs from n-3 PUFAs possess anti-inflammatory properties, which may partly explain the lower incidences of diseases when supplemental n-3-rich oils are provided [8]. However, the effects of adding n-3 FAs to SF or milk replacers for pre-weaning calves have yielded mixed results. Ballou and DePeters [9] reported that supplementing fish oil did not lead to an improved health status in Jersey calves, although they observed a dose-dependent effect of fish oil on different immune responses. Another study [10] showed that an altered n-6/n-3 FA ratio can affect DMI and animal performance. An increased daily gain and feed efficiency has been reported in calves fed a flax oil-supplemented diet compared with fish oil-supplemented diet, with no difference compared to calves fed the control diet [11]. In a recent study, calves fed fish oil compared with canola oil had greater daily gain in the pre-weaning period, greater DMI during last 10 days of the rearing period, and a modulated pattern of inflammatory mediators [12]. Apart from the level of fat supplementation, the type of FAs and the ratio of n-3 to other FAs appear to be significant dietary factors contributing to varying results in different studies [13]. In contrast to n-3 FAs, n-6 FAs are involved in producing pro-inflammatory mediators [14]. Goldman et al. [15] revealed that a portion of the anti-inflammatory characteristics of n-3 FAs is related to their competition with n-6 series for the substrates needed to metabolize n-6 FAs. Therefore, the composition of FAs in animal diets may be a critical factor in regulating inflammatory responses and, consequently, have carryover effects on animal growth and health [16]. Studies in humans have shown that the dietary ratio of n-6 to n-3 FAs is directly linked to an increased risk of inflammation-related diseases [17]. This factor may also play a crucial role in the growth and metabolic performance of newborn calves.

In line with this, the present study hypothesizes that reducing the n-6/n-3 ratio in SF by incremental levels of n-3 FAs will enhance energy intake, metabolic status, and growth performance in young calves. To achieve the reduction of the n-6/n-3 ratio, fish oil was employed, as it is not only a rich source of n-3 FAs but also contains eicosapentaenoic (EPA) and docosahexaenoic acid (DHA), with immune-modulating effects that are absent in plant oils [18]. The increased availability of eicosanoids with inflammation-resolving properties is expected to enhance the anti-inflammatory capacity [1]. Therefore, the objective of this study was to determine the effect of a decreasing n-6/n-3 FA ratio in SF on the metabolic status, ruminal fermentation characteristics, eating behavior indexes, and growth performance of pre-weaning Holstein calves. The gradual decrease in the n-6/n-3 ratio was achieved by partial or complete replacement of palm fatty acids with fish oil.

## 2. Materials and Methods

The experiment was carried out in a commercial dairy farm in Alborz, Iran. The Animal Care Committee of University of Zanjan (ID 1353) approved the procedure related to animal care and management.

### 2.1. Animals, Treatments, and Management

**Figure 1:**
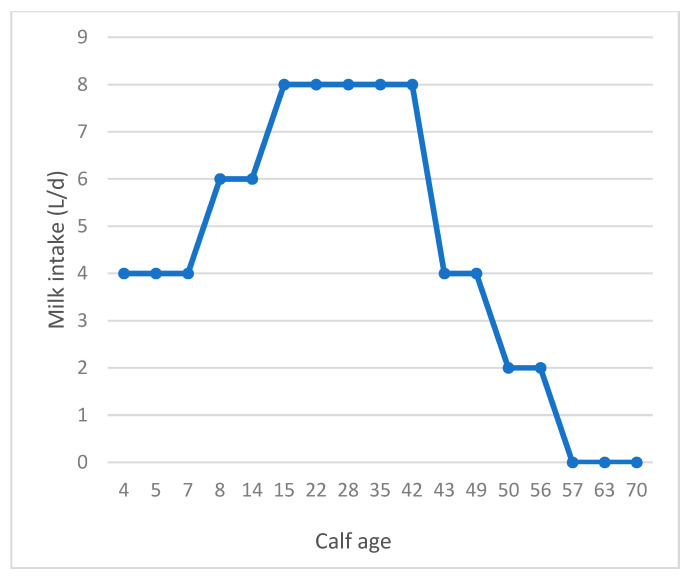
Milk intake (L/d) in calves fed with whole milk. Weaning was performed on day 57.

Thirty female calves were selected and weighed immediately after birth (birth body weight (BBW) of 39.4 ± 3.1) and housed in individual pens (1.2 × 2.5 m^2^) with straw bedding. Within the first 2 to 3 h of life, 4 L of pooled colostrum were fed to all calves, and the transition milk feeding was continued until 3 days of age. From day 4 to day 56 of age, all animals received whole milk (containing 3.55% ± 0.34 milk fat, 3.18% ± 0.11 crude protein (CP), 4.65% ± 0.03 lactose, and 11.60% ± 0.11 total solid content) twice a day (at 0800 and 1600) through buckets. Calves at the age of 4 to 7 d, 8 to 14 d, 15 to 42 d, 43 to 49 d, and 50 to 56 d received 4, 6, 8, 4, and 2 L of milk, respectively (Figure 1). All calves were weaned at day 57 of age; however, the study continued until day 70 of life (Figure 1). From the age of 3 d, calves were fed one of the three dietary treatments: (1) SF supplemented with 3.3% palm fatty acids (PO), (2) SF supplemented with 1.7% of palm fatty acids and 1.9% calcium salt of fish oil (PFO), or (3) SF supplemented with 3.9% calcium salt of fish oil (FO). Incremental replacement of palm fatty acids with fish oil resulted in n-3 FA levels (in % of total FA) of 1.90, 6.80, and 11.8 and in n-6/n-3 ratios of 15.5, 4.50, and 2.70 for PO, PFO, and FO, respectively. The prilled palm fatty acids and calcium salt of fish oil were mixed as solid powders with concentrate; mixtures were stored in a cold place for use over one week. Chopped straw at 7.5% (DM) was thoroughly mixed into the supplemented SF of all treatments to create a total mixed ration (TMR). Diets were formulated using CPM dairy (V. 3.1.0.7) to meet nutritional requirements for young calves. Nutrient compositions of the ingredients and their contents in the starter diets are presented in Table 1. The fatty acids profile of FO and PO was determined using gas chromatography (Table 2). Starter feed at least 10% higher than calves’ individual appetites was provided (i.e., ad libitum), and the orts of all individuals were collected at 15:00. Animals had free access to fresh water at all times.

### 2.2. Sampling

Starter feed intake (starter plus forage as TMR) and total dry matter intake (DMI) were calculated daily. Calves BW during pre-weaning (week 1 to 8), post weaning (week 9 and 10), and the entire period (week 1 to 10) were recorded on a weekly basis. The weights of the offered feed and orts were recorded daily for each calf. Average DMI, average daily gain (ADG), and ratio of daily gain to feed intake (G:FI) were recorded for pre- and post-weaning periods and the entire period.

Body size parameters, including body length, body girth, withers height, heart girth, hip height, and hip width, were determined at the ages of 1, 19, 57, and 70 d, according to Khan et al. [19].

Behavioral data were measured as direct observation of all calves and collected as assigned time (min) per behavioral parameter over 4 d during a 2-week pre-weaning (age 40 and 47 d) and 2-week post-weaning period (age 61 and 68 d). During pre-weaning, the parameters of calves’ behavior were recorded over 1 h before offering initial feed and for the first 2 h after offering initial feed. Therefore, the collective time of investigating each calf’s behavior was 12 h (3 h/d for 4 d). Each observational period was split into 5-min intervals in which the following behaviors were recorded: standing, laying, ruminating, and non-eating oral behaviors; zero behavior was registered when none of mentioned behaviors could be observed.

Calves’ feces were scored (1 = thick to 5 = watery, mucous, and bloody) for its physical appearance and consistency, according to Heinrichs et al. [20].

On days 42 and 70 of the study, ruminal fluid samples were taken using a stomach tube 4 h after the evening feeding. Immediately after taking the sample, ruminal pH was determined (pH Pen, 8686 AZ, AZ Instrument Co, Taichung City, Taiwan). Then, an 8 mL ruminal fluid sample was immediately acidified with 2 mL of metaphosphoric acid (25%) and stored at −20 °C for analysis of short chain fatty acid (SCFA) [21].

Blood samples were taken from the jugular vein 3 h after the evening feeding on days 15, 43, and 70 of the experiment. Samples were taken using evacuated tubes coated with potassium–ethylenediamine tetra-acetic acid (EDTA/K3) with a concentration of 1.27 mg EDTA/K3 per ml of blood for plasma metabolites and hematology analysis. The first aliquot of samples was centrifuged at 3000× *g* for 15 min to obtain plasma. Plasma samples were stored at −20 °C for later laboratory analysis of glucose, urea, total protein, albumin, and beta-hydroxybutyrate (BHB) using an auto analyzer (Technicon-RA 1000 Autoanalyzer; DRG Instruments GmbH, Marburg, Germany) and relevant commercial kits (Pars Azmun Laboratory, Tehran, Iran). A second aliquot of blood samples was stored on ice, transported to the veterinary laboratory, and then placed in a water bath at 37 °C for 30 min or brought to room temperature for hematology analysis.

The dry matter values of feed and orts samples were analyzed according to AOAC [22] to evaluate crude protein (method 988.05), ether extract (method of 920.39), and ash (method of 942.05) of feed samples and refusals. Milk samples were collected weekly. The pooled samples of two consecutive weeks were analyzed for fat, protein, lactose, and total solid content (Milko Scan 4000, Foss Electric, Hillerod, Denmark).

### 2.3. Statistical Analysis

Data were analyzed using the MIXED procedure of SAS 9.4 (SAS Institute Inc., Cary, NC, USA). Data were tested for normality by using PROC UNIVARIATE. The statistical model included the fixed effects of diet, time of measurement and their interactions and the random effect of the calf and residuals. Data were analyzed separately for pre-weaning (weeks 1 to 8) and post-weaning (weeks 9 to 10) periods and the overall period (weeks 1 to 10). Repeated-measures analysis was used for intakes of starter feed as well as total DMI (recorded daily), BW, ADG, and gain to feed ratio (recorded weekly); skeletal growth (days 1, 19, 57, and 83); ruminal pH; ruminal metabolites; and blood parameters over time. Initial body weight and structural growth measurements were considered as covariates. A heterogeneous autoregressive type 1 covariance structure was used in the mixed model based on the lowest Akaike’s information criterion. If the primary test indicated statistical significance, differences among treatment means were determined using Tukey’s multiple-range tests. Sums of squares for treatment effects were further separated using orthogonal contrasts into a single degree-of-freedom comparison to test for the significance of linear and quadratic components of the response to incremental levels of n-3 fatty acids by replacing PO with FO. Least-square means are reported, and treatment effects were declared significant at *p* < 0.05, with *p*-values between 0.05 and 0.10 being considered a trend towards significance.

## 3. Results

### 3.1. Weekly BW, ADG, Starter DMI, Total DMI, and G:FI

**Figure 2:**
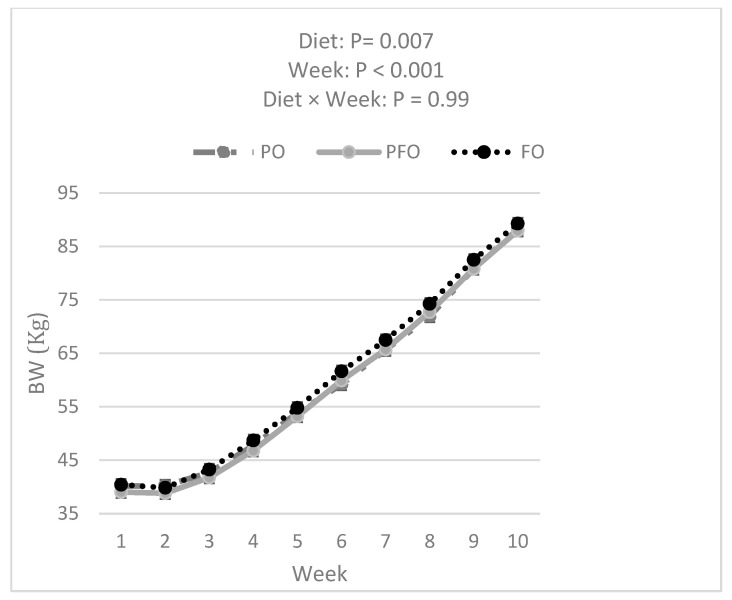
Body weights (BW) for calves fed supplemental oils. Treatments consisted of starter diets containing 3.3% palm fatty acids (PO), 3.9% calcium salt fish oil (FO), or 1.7% palm fatty acids and 1.9% calcium salt fish oil (PFO).

**Figure 3:**
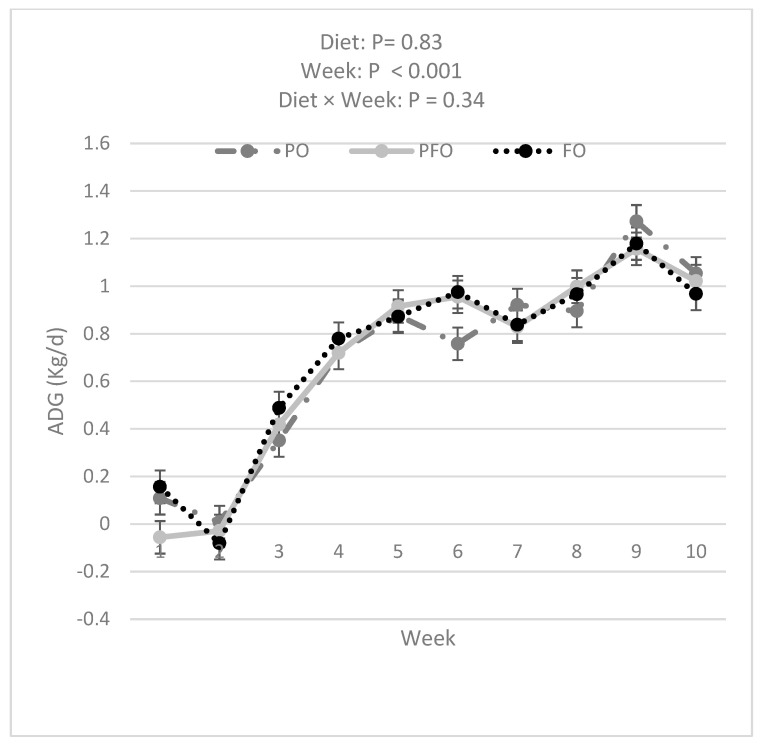
Average daily gain (ADG) for calves fed supplemental oils. Treatments consisted of starter diets containing 3.3% palm fatty acids (PO), 3.9% calcium salt fish oil (FO), or 1.7% palm fatty acids and 1.9% calcium salt fish oil (PFO).

**Figure 4:**
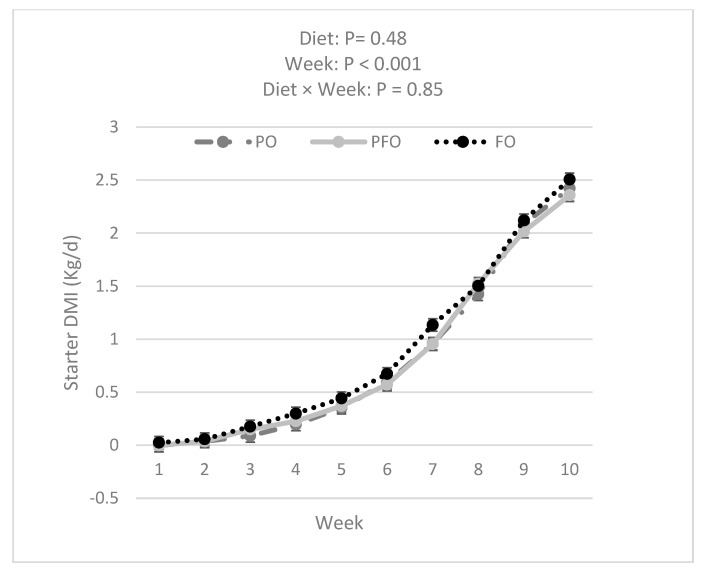
Starter dry matter intake (DMI) for calves fed supplemental oils. Treatments consisted of starter diets containing 3.3% palm fatty acids (PO), 3.9% calcium salt fish oil (FO), or 1.7% palm fatty acids and 1.9% calcium salt fish oil (PFO).

**Figure 5:**
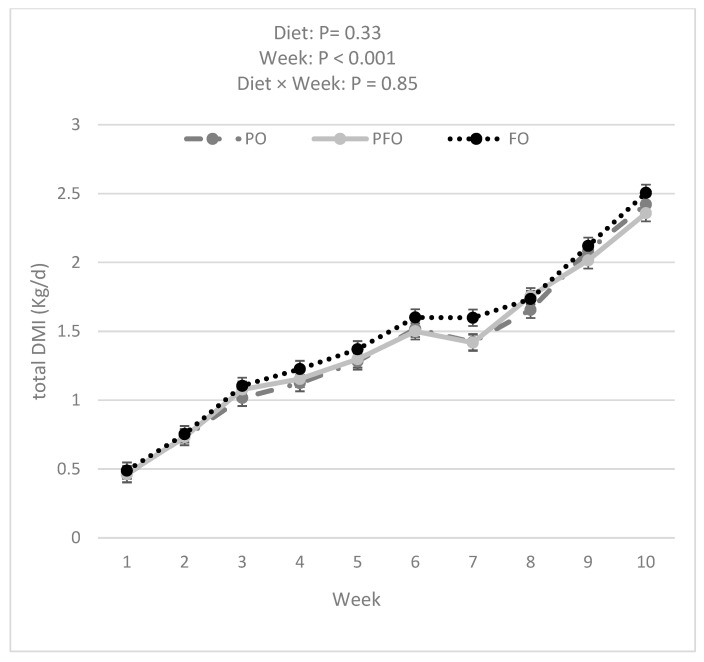
Total dry matter intake (DMI) for calves fed supplemental oils. Treatments consisted of starter diets containing 3.3% palm fatty acids (PO), 3.9% calcium salt fish oil (FO), or 1.7% palm fatty acids and 1.9% calcium salt fish oil (PFO).

**Figure 6:**
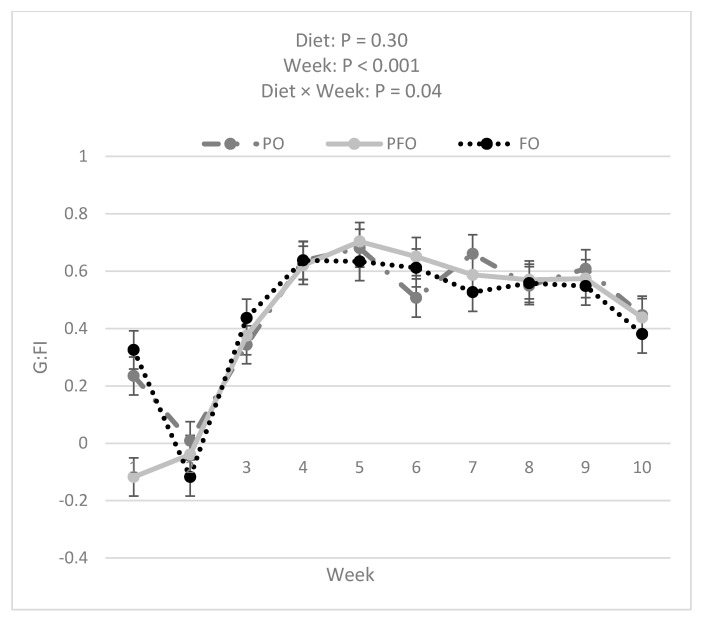
Gain to feed intake ratio (G:FI) for calves fed supplemental oils. Treatments consisted of starter diets containing 3.3% palm fatty acids (PO), 3.9% calcium salt fish oil (FO), or 1.7% palm fatty acids and 1.9% calcium salt fish oil (PFO).

Data analysis showed that FO calves had a greater BW compared with PFO (*p =* 0.007) and tended to have greater BW than PO (*p =* 0.050) calves (60.2 ± 0.3, 58.7 ± 0.3, and 59.0 ± 0.3 for FO, PFO, and PO calves, respectively) during the entire experimental period (Figure 2). The BW of all calves increased over the 10 weeks of the experiment (Figure 2). However, BW was not influenced by the interaction effect of diet × week (*p =* 0.99). PFO-fed calves showed lower ADG than PO- or FO-fed animals in weeks 1 and 6; ADG during the 10 weeks of study did not differ among dietary treatments (*p =* 0.83, Figure 3). Starter and total DMI were not changed by dietary treatments across the entire experimental period (*p =* 0.33; Figure 4 and Figure 5). An interaction effect of diet × time was observed only for G:FI ratio (*p =* 0.04), with a lower G:FI in the PFO group compared to the other dietary groups in week 1 (*p =* 0.0016; Figure 6). Interaction effects of diet × time were not significant for all other variables (*p* > 0.1)

### 3.2. Growth Indices, Blood Variables, and Ruminal Fermentation Characteristics

Dietary treatments did not impact growth indices such as body length, body girth, withers height, heart girth, hip height, and hip width, except for temporarily higher body girth, withers heights, and hip heights in PFO calves compared with PO and FO calves on day 43 (Table 3). Neither ruminal fermentation parameters (Table 4) nor blood variables, the latter including blood metabolites and immune system biomarkers (Table 5), were influenced by adding different types of oil (*p* > 0.05).

### 3.3. Animal Behavioral Parameters and Calf Scour

The results of behavioral parameters are presented in Table 6. Data analysis showed that behavioral parameters such as standing, lying, eating, and ruminating were not influenced by different dietary treatments (*p* > 0.05). The number of days with abnormal fecal score was not different among different dietary groups (*p* > 0.05).

## 4. Discussion

Opposite to our hypothesis, adding fish oil to SF to change the n-6/n-3 ratio of the supplemental oils (from 15.5 to 4.50 and finally 2.70 for FPO, PFO, and FO diets, respectively) did not lead to improved growth and metabolic performance or any changes in ruminal fermentation and animal eating behavior. One reason for these results may be related to the low amount of actual PUFA intake by the calves. For weeks 1 to 4, the average fish oil fed to the FO group was 0.50, 1.8, 6.4, and 11 g of fish oil, respectively. The corresponding amounts for PFO were 0.11, 0.74, 2.6, and 3.9 g of fed fish oil, calculated based on starter DMI and the percentage of fish oil in SF. Therefore, the low amount of n-3 in SF may have hardly changed the n-6/n-3 ratio of the whole diet to induce any subsequent metabolic changes. To date, no optimal amount of n-3 FA or n-6/n-3 ratios for feeding have been suggested for newborn calves’ diets. Attempts to alter the n-6/n-3 ratio of the diet have primarily focused on meat quality [23,24]. However, data are limited regarding the effects of altering n-6/n-3 in SF of growing calves on ruminal fermentation, metabolic status, and eating behavior variables.

Although contrasting our hypothesis, the results of the current study are consistent with other studies where higher amounts of n-3 PUFA from fish oil or different vegetable oils did not impact DMI and growth performance in growing lambs [25], calves [9], and goats [24]. We monitored different growth performance parameters, including BW, starter DMI, total DMI, ADG, and G:FI. We observed a main effect of diet on BW. However, as this was not accompanied by a diet × time interaction, it was not a consequence of the different oil supplementations but already present at the start of the experiment. The only interaction between diet × time was observed for G:FI with lower values in the PFO group during the first week of experiment. This may lead to the general conclusion that the changes in the n-6/n-3 ratio, when extrapolated to the whole diet, were possibly not large enough to induce changes in ruminal fermentation patterns or nutrient partitioning. Some previous research also reported that the type of FA did not have a major effect on the growth performance of growing ruminants when the amount of energy was similar among dietary treatments [23,26]. Ghasemi et al. [27] reported no effect on calf DMI when supplementing different types of fat, such as palm vs. soybean oils (the latter as a source of PUFA).

Nonetheless, some reports have shown that an altered n-6/n-3 FA ratio can affect DMI and animal performance [10]. However, results are partly divergent. Panahiha et al. [6,7] showed that performance improved by supplementing palm fatty acids compared with soybean oil in the starter diet. In contrast, Hill et al. [3,4] showed that ADG increased by adding sources of n-3 PUFA (C18:2 and C18:3) to the SF in calves up to 120 days of life. When interpreting studies that reported enhanced growth performance in PUFA-supplemented calves, one has to consider that supplemented animals had often a greater energy intake compared to non-supplemented ones; i.e., the impact of feeding extra energy can confound the effect of adding PUFA [4,28]. The latter does not apply to the current experiment where all diets were formulated to have similar energy and protein contents.

Pre-weaning calves are not developed ruminants, and ruminal biohydrogenation does not occur for supplemental FA in their milk replacer or SF [28]. Therefore, PUFA addition may have non- or only minor effects on ruminal fermentation, and secondly, the bioavailability of supplemental FA should be higher in newborn ruminants [28]. Considering no alteration in ruminal fermentation patterns among different dietary groups, it is not surprising that no difference in total intakes of DM or SF was observed among animals in the present study. Moreover, the lack of significant change in ruminal fermentation patterns and energy status biomarkers (e.g., BHB and glucose) among treatments shows that all animals likely had a similar energetic/metabolic balance as well. Studies showing a positive effect of fat supplements mostly compared diets with high and low or no added fat, and the application of FA may spare the utilization of other energy substrates such as glucose [8,27]. Therefore, our results confirm previous studies reporting that the source of energy is a less important factor when ruminant animals receive similar energy and protein levels [29].

The known anti-inflammatory effect of PUFA in fish oil did not alter leucocyte counts and compositions as immune-related variables in the present study. These results were accompanied by no change in the number of days with diarrhea in young calves among different dietary groups. Such results are consistent with other studies that did not find a positive impact of adding fish oil on diarrhea in calves [9,11].

## 5. Conclusions

The newborn Holstein calves of the present study did not benefit from adding a rich source of n-3 PUFA to SF during the first 70 days of life under our experimental conditions. This was indicated by similar growth and metabolic, ruminal, and eating behavior variables. It seems that the supplement of different types of FA at a very moderate level of ~3–4% of DM does not affect the growth or metabolic performance of newborn calves when diets have similar FA and energy contents. This does not negate that supplemental PUFA may have beneficial effects at higher concentrations or during immunological or metabolic challenge periods. Therefore, further studies are needed to test higher levels of n-3 FA in calves’ diets in normal and challenging situations.

## Figures and Tables

**Table 1:** Formulation of experimental starter diets (% of DM) with palm fatty acids and calcium salt of fish oil.

	Diets ^1^		
Item	PO	PFO	FO
Ground barley grain	15.9	15.9	16.0
Ground corn grain	42.5	42.5	42.5
Wheat bran	4.50	4.80	4.90
Soybean meal	24.7	24.6	24.5
Extruded soybean	1.60	1.60	1.60
Corn gluten meal	1.60	1.60	1.60
Prilled palm fatty acids ^2^	3.30	1.70	0.00
Fish oil (calcium salt) ^3^	0.00	1.90	3.90
Sodium bicarbonate	1.30	1.30	1.30
Salt	0.40	0.40	0.40
Calcium carbonate	0.90	0.40	0.00
Minerals and vitamins ^4^	2.00	2.00	2.00
Sodium bentonite	1.30	1.30	1.30
**Chemical composition**			
Dry matter (DM)	91.0	91.0	91.0
Crude protein (CP)	18.6	18.6	18.6
Ether extract (EE)	6.60	6.60	6.60
Neutral detergent fiber (NDF)	12.6	12.7	12.8
Non-fiber carbohydrate ^5^	54.2	54.3	54.4
Ash	9.10	8.90	8.70

^1^ Treatments consisted of starter diets containing 3.3% palm fatty acids (PO), 3.9% calcium salt fish oil (FO), or 1.7% palm fatty acids and 1.9% calcium salt fish oil (PFO). Chopped straw (7.5% of DM) was included in the starter feed of all treatments. ^2^ Consisting of 75% C16:0, 5% C18:0, 15% C18:1, 4% C18:2, and 1% others (values are % of total fatty acids). ^3^ Consisting of 8.2% C14:0, 16.5% C16:0, 9.6% C16:1, 3.7% C18:0, 13% C18:1, 1.4% C18:2, 3% C 18:3, 0.5% C20:0, 11.6% C20:5, 10.4% C22:6, and 22.2% others (values are % of total fatty acids). ^4^ Containing (per kg) 300,000 IU vit A, 25,000 IU vit D3, 2000 IU vit E, 210 g Ca, 15 g Mg, 1.8 g Zn, 0.4 g Cu, 2.2 g Mn, 15 mg Co, 15 mg iodine, and 10 mg Se. ^5^ NFC = 100 − (% NDF + % CP + % EE + % Ash).

**Table 2:** Profile of fatty acid in starter feed in different dietary treatments (% of total fatty acid).

	Diets ^1^		
Name	PO	PFO	FO
C12:0	0.00	1.00	2.10
C14:0	0.70	0.90	1.20
C16:0	44.2	31.1	17.2
C16:1	0.00	1.30	2.70
C18:0	3.70	6.30	8.90
C18:1	18.1	20.6	23.3
C18:2	29.4	30.7	32.3
C18:3	1.90	3.20	4.50
C20:5 + C22:6	0.0	3.60	7.30
Other FA	2.00	1.20	0.40
Total FA	100	100	100
Total n-3 FA	1.90	6.80	11.8
n-6/n-3	15.5	4.50	2.70

^1^ Treatments consisted of starter diets containing 3.3% palm fatty acids (PO), 3.9% calcium salt fish oil (FO), or 1.7% palm fatty acids and 1.9% calcium salt fish oil (PFO).

**Table 3:** Effects of incremental replacement of palm fatty acids with fish oil in starter feed on skeletal growth indices of Holstein calves.

	Diet ^1^					*p*-Value			
Item	PO	PFO	FO	SEM	Diet	Linear	Quadratic	Diet × Time	Time
Body length, cm									
(days 3–70)	89.6	86.9	89.3	2.3	0.44	0.93	0.21	0.41	<0.0001
(day 3)	77.9	76.1	77.6	4.1	0.65	0.25	0.21		
(day 43)	90.7 ^a^	79.8 ^b^	87.9 ^ab^	4.2	0.02	0.30	0.24		
(day 56)	92.0	93.3	93.5	4.1	0.74	0.93	0.21		
(day 70)	97.7	98.3	98.3	4.1	0.96	0.30	0.22		
Body girth, cm									
(days 3–70)	104.4	101.0	104.5	2.4	0.26	0.97	0.11	0.37	<0.0001
(day 3)	78.7	77.4	78.4	4.7	0.78	0.16	0.11		
(day 43)	102.3 ^a^	91.1 ^b^	104.3 ^a^	4.8	0.03	0.16	0.12		
(day 56)	112.7	111.8	112.7	4.7	1.00	0.97	0.11		
(day 70)	124.0	123.6	122.6	4.7	0.83	0.16	0.11		
Withers height, cm									
(days 3–70)	85.5	82.4	85.0	2.0	0.26	0.78	0.11	0.33	<0.0001
(day 3)	77.2	76.6	77.2	3.6	0.86	0.13	0.11		
(day 43)	86.2 ^a^	75.2 ^b^	83.4 ^a^	3.6	0.03	0.22	0.15		
(day 56)	87.5	86.6	87.9	3.6	0.72	0.78	0.11		
(day 70)	91.3	91.3	91.3	3.6	0.99	0.22	0.13		
Heart girth, cm									
(days 3–70)	92.1	89.3	92.6	2.1	0.24	0.84	0.098	0.73	<0.0001
(day 3)	77.4	76.9	76.9	4.0	0.89	0.17	0.10		
(day 43)	90.7 ^ab^	82.7 ^a^	91.8 ^b^	4.1	0.03	0.13	0.10		
(day 56)	97.1	95.4	97.9	4.0	0.53	0.84	0.10		
(day 70)	103.4	102.2	103.7	4.0	0.71	0.13	0.10		
Hip height, cm									
(days 3–70)	89.4	86.4	89.4	2.0	0.24	0.99	0.10	0.43	<0.0001
(day 3)	80.8	79.8	81.2	3.8	0.79	0.14	0.10		
(day 43)	88.9 ^a^	78.9 ^b^	88.5 ^a^	3.9	0.01	0.15	0.11		
(day 56)	92.7	91.2	92.7	3.8	0.69	1.00	0.10		
(day 70)	95.1	95.7	95.3	3.8	0.97	0.15	0.10		
Hip width, cm									
(days 3–70)	20.0	20.2	20.2	0.3	0.78	0.51	0.83	0.53	<0.0001
(day 3)	17.3	17.3	17.1	0.4	0.98	0.60	0.83		
(day 43)	19.6	19.6	19.8	0.4	0.85	0.88	0.98		
(day 56)	21.1	21.2	21.2	0.4	0.80	0.51	0.83		
(day 70)	22.2	22.7	22.8	0.4	0.80	0.88	0.91		

^1^ Treatments consisted of starter diets containing 3.3% palm fatty acids (PO), 3.9% calcium salt fish oil (FO), or 1.7% palm fatty acids and 1.9% calcium salt fish oil (PFO). ^a,b^ Different superscripts within rows indicate significant differences (*p* < 0.05).

**Table 4:** Effects of incremental replacement of palm fatty acids with fish oil in starter feed on ruminal fermentation characteristics of Holstein calves.

	Diets ^1^					*p*-Value			
Item	PO	PFO	FO	SEM	Diet	Linear	Quadratic	Diet × Time	Time
Rumen pH									
Entire period	5.98	6.05	5.98	0.19	0.91	0.98	0.67	0.38	0.024
Pre-weaning period (day 42)	5.96	5.84	5.80	0.24	0.62	0.72	0.67		
Post-weaning period (day 70)	6.01	6.27	6.16	0.24	0.53	0.70	0.67		
Total SCFA, m*M*									
Entire period	38.5	39.8	40.3	4.7	0.93	0.71	0.91	0.56	0.009
Pre-weaning period (day 42)	35.8	32.4	35.4	6.5	0.95	0.78	0.91		
Post-weaning period (day 70)	41.3	47.3	45.2	6.3	0.74	0.93	0.100		
Acetate, mol/100 mol SCFA									
Entire period	47.9	48.4	48.4	1.3	0.92	0.73	0.84	0.77	0.78
Pre-weaning period (day 42)	48.0	48.5	47.8	1.8	0.77	0.73	0.84	0.84	
Post-weaning period (day 70)	47.8	48.2	49.0	1.7	0.51	1.00	0.92	0.92	
Propionate, mol/100 mol SCFA									
Entire period	33.6	33.2	31.9	1.3	0.43	0.22	0.68	0.69	0.0008
Pre-weaning period (day 42)	31.6	30.7	30.5	2.0	0.61	0.78	0.68		
Post-weaning period (day 70)	35.6	35.7	33.2	1.9	0.96	0.33	0.46		
Butyrate, mol/100 mol SCFA									
Entire period	10.4	10.3	11.3	0.8	0.42	0.28	0.47	0.95	<0.0001
Pre-weaning period (day 42)	11. 7	11.7	12.8	1.1	0.94	0.96	0.47		
Post-weaning period (day 70)	9.1	8.9	9.8	1.0	0.42	0.24	0.32		
Valerate, mol/100 mol SCFA									
Entire period	5.06	5.05	5.24	0.44	0.87	0.66	0.79	0.80	0.047
Pre-weaning period (day 42)	5.62	5.44	5.50	0.70	0.87	0.99	0.79		
Post-weaning period (day 70)	4.49	4.65	4.98	0.72	0.80	0.65	0.70		
Isovalerate, mol/100 mol SCFA									
Entire period	3.08	3.04	3.18	0.31	0.90	0.79	0.72	0.47	0.029
Pre-weaning period (day 42)	3.22	3.61	3.44	0.51	0.72	0.86	0.72		
Post-weaning period (day 70)	2.94	2.48	2.94	0.50	0.34	0.65	0.67		
Acetate/propionate ratio									
Entire period	1.45	1.48	1.64	0.16	0.48	0.26	0.68	0.93	0.085
Pre-weaning period (day 42)	1.53	1.60	1.79	0.24	0.78	0.82	0.68		
Post-weaning period (day 70)	1.36	1.37	1.49	0.23	0.97	0.35	0.47		

^1^ Treatments consisted of starter diets containing 3.3% palm fatty acids (PO), 3.9% calcium salt fish oil (FO), or 1.7% palm fatty acids and 1.9% calcium salt fish oil (PFO).

**Table 5:** Effects of incremental replacement of palm fatty acids with fish oil in starter feed on blood metabolites and hematology parameters of Holstein calves.

	Diet ^1^					*p*-Value			
Item	PO	PFO	FO	SEM	Diet	Linear	Quadratic	Diet × Time	Time
Glucose, mg/dL									
Entire period	87.4	97.0	94.1	4.3	0.084	0.12	0.100	0.73	<0.001
Pre-weaning period (day 15)	94.3	109.0	99.9	7.6	0.47	0.030	0.100		
Pre-weaning period (day 43)	95.2	107.0	104.8	7.4	0.77	0.51	0.24		
Post-weaning period (day 71)	72.7	74.9	77.7	7.4	0.71	0.12	0.100		
Total protein, g/dL									
Entire period	6.73	6.80	6.67	0.20	0.79	0.76	0.54	0.42	0.20
Pre-weaning period (day 15)	6.70	6.91	6.43	0.26	0.42	0.70	0.54		
Pre-weaning period (day 42)	6.74	6.72	6.64	0.25	0.94	0.49	0.50		
Post-weaning period (day 70)	6.74	6.78	6.93	0.25	0.55	0.76	0.54		
Albumin, g/dL									
Entire period	3.49	3.44	3.50	0.06	0.61	0.89	0.32	0.96	<0.001
Pre-weaning period (day 15)	3.12	3.06	3.13	0.11	0.59	0.44	0.32		
Pre-weaning period (day 42)	3.74	3.71	3.70	0.11	0.79	0.35	0.32		
Post-weaning period (day 70)	3.61	3.55	3.67	0.11	0.59	0.89	0.32		
BHBA, mmol/L									
Entire period	0.33	0.28	0.29	0.04	0.42	0.28	0.45	0.99	<0.001
Pre-weaning period (day 15)	0.21	0.15	0.17	0.06	0.50	0.23	0.45		
Pre-weaning period (day 42)	0.27	0.25	0.24	0.06	0.64	0.91	0.67		
Post-weaning period (day 70)	0.50	0.46	0.46	0.06	0.46	0.28	0.45		
Total leucocytes, G/L									
Entire period	12.0	10.9	11.9	1.0	0.52	0.91	0.26	0.90	0.001
Pre-weaning period (day 15)	13.8	12.6	13.1	1.5	0.65	0.30	0.26		
Pre-weaning period (day 42)	10.4	9.3	11.2	1.5	0.56	0.36	0.29		
Post-weaning period (day 70)	11.9	10.8	11.3	1.5	0.71	0.91	0.26		
Neutrophils, %									
Entire period	36.8	34.3	37.2	2.9	0.56	0.90	0.29	0.71	0.19
Pre-weaning period (day 15)	42.0	36.8	37.6	4.9	0.38	0.39	0.29		
Pre-weaning period (day 42)	36.2	34.7	36.4	4.8	0.97	0.32	0.29		
Post-weaning period (day 70)	32.2	31.4	37.5	4.8	0.80	0.90	0.29		
Lymphocytes, %									
Entire period	57.2	60.1	56.9	2.9	0.47	0.91	0.22	0.83	0.21
Pre-weaning period (day 15)	52.7	57.9	56.2	5.0	0.72	0.32	0.22		
Pre-weaning period (day 42)	57.3	59.3	57.3	4.8	0.68	0.26	0.23		
Post-weaning period (day 70)	61.6	63.1	57.1	4.8	0.76	0.91	0.22		
Monocytes, %									
Entire period	3.7	3.3	3.4	0.2	0.15	0.13	0.21	0.36	0.007
Pre-weaning period (day 15)	3.2	3.0	3.2	0.3	0.90	0.07	0.21		
Pre-weaning period (day 42)	3.8	3.5	3.7	0.3	0.74	0.74	0.43		
Post-weaning period (day 70)	4.0	3.4	3.2	0.3	0.51	0.13	0.21		
Eosinophils, %									
Entire period	2.4	2.4	2.6	0.1	0.38	0.20	0.60	0.11	0.19
Pre-weaning period (day 15)	2.1	2.3	2.9	0.3	0.48	0.84	0.60		
Pre-weaning period (day 42)	2.7	2.5	2.6	0.3	0.70	0.27	0.39		
Post-weaning period (day 70)	2.3	2.4	2.2	0.3	0.70	0.20	0.60		

^1^ Treatments consisted of starter diets containing 3.3% palm fatty acids (PO), 3.9% calcium salt fish oil (FO), or 1.7% palm fatty acids and 1.9% calcium salt fish oil (PFO).

**Table 6:** Effects of incremental replacement of palm fatty acids with fish oil in starter on activities (standing or lying) and eating behaviors of dairy calves (min/12 h observation).

	Diet ^1^					*p*-Value			
Item	PO	PFO	FO	SEM	Diet	Linear	Quadratic	Diet × Time	Time
Standing									
Entire period	89.9	91.2	78.6	7.23	0.18	0.13	0.27	0.52	0.26
day 40	95.0	96.0	82.5	10.0	0.92	0.85	0.27		
day 47	89.5	87.0	79.0	10.0	0.80	0.092	0.14		
day 61	95.5	95.5	72.5	10.0	1.00	0.13	0.27		
day 68	79.5	86.5	80.5	10.0	0.55	0.091	0.19		
Lying									
Entire period	90.1	88.7	101	7.23	0.18	0.14	0.27	0.52	0.26
day 40	85.0	84.0	97.5	10.1	0.92	0.85	0.27		
day 47	90.5	93.0	101	10.1	0.80	0.092	0.14		
day 61	84.5	84.5	107	10.1	1.00	0.13	0.27		
day 68	100	93.5	99.5	10.1	0.92	0.091	0.19		
Eating									
Entire period	30.9	29.9	29.1	3.63	0.89	0.63	0.97	0.22	<0.0001
day 40	15.5	15.5	21.0	6.52	1.00	0.78	0.97		
day 47	28.0	31.5	34.0	6.52	0.59	0.84	0.92		
day 61	46.0	38.5	30.0	6.52	0.25	0.63	0.97		
day 68	34.0	34.0	31.5	6.52	0.70	0.84	0.98		
Non-nutritional oral behaviors									
Entire period	43.9	40.4	32.2	5.76	0.14	0.053	0.65	0.45	0.23
day 40	44.0	44.5	36.0	7.85	0.95	0.55	0.65		
day 47	46.0	36.0	24.5	7.85	0.21	0.17	0.32		
day 61	44.0	35.5	31.5	7.85	0.61	0.052	0.65		
day 68	41.5	45.5	37.0	7.85	0.57	0.17	0.46		
Ruminating									
Entire period	28.4	30.6	31.0	4.67	0.83	0.58	0.82	0.73	0.12
day 40	28.0	37.5	33.0	7.91	0.53	0.63	0.82		
day 47	36.5	32.5	34.0	7.91	0.61	0.94	0.95		
day 61	20.5	24.5	31.0	7.91	0.41	0.58	0.82		
day 68	28.5	28.0	26.0	7.91	0.80	0.94	0.89		
Idle									
Entire period	76.9	79.1	87.6	5.67	0.15	0.068	0.53	0.71	0.33
day 40	92.5	82.5	90.0	11.0	0.82	0.69	0.53		
day 47	69.5	80.0	87.5	11.0	0.49	0.14	0.26		
day 61	69.5	81.5	87.5	11.0	0.58	0.071	0.53		
day 68	76.0	72.5	85.5	11.0	0.75	0.14	0.37		

^1^ Treatments consisted of starter diets containing 3.3% palm fatty acids (PO), 3.9% calcium salt fish oil (FO), or 1.7% palm fatty acids and 1.9% calcium salt fish oil (PFO).

## Data Availability

The data presented in this study are available on request from the corresponding author.
